# Genomic Signature in Evolutionary Biology: A Review

**DOI:** 10.3390/biology12020322

**Published:** 2023-02-16

**Authors:** Rebeca de la Fuente, Wladimiro Díaz-Villanueva, Vicente Arnau, Andrés Moya

**Affiliations:** 1 Institute of Integrative Systems Biology (I2Sysbio), University of Valencia and Spanish Research Council (CSIC), 46980 Valencia, Spain; 2 Foundation for the Promotion of Sanitary and Biomedical Research of the Valencian Community (FISABIO), 46020 Valencia, Spain; 3 CIBER in Epidemiology and Public Health (CIBEResp), 28029 Madrid, Spain

**Keywords:** genomic signature, chaos game representation, genome sequence, alignment-free methods, evolutionary biology

## Abstract

**Simple Summary:**

In a broad sense, genomic signature refers to characteristics associated to DNA sequences. Many studies analyze genotype–phenotype patterns in a group of genes, thus targeting genomic signatures associated to a given disease or identifying a gene expression profile. However, some studies in comparative genomics and evolutionary biology refer to genomic signature as the statistical properties of DNA sequences, such as the distribution of k-words. In these fields of study, genomic signatures are species-specific and can be informative about phylogenetic relationships. In this review, we identify the main genomic signatures in a large collection of articles by performing a bibliometric analysis and then rename each signature according to its conceptual meaning. Among the different signatures, we use the term *organismal signature* to denote the DNA patterns able to infer evolutionary relationships and go on to review its formulation and applications in the second part of the article.

**Abstract:**

Organisms are unique physical entities in which information is stored and continuously processed. The digital nature of DNA sequences enables the construction of a dynamic information reservoir. However, the distinction between the hardware and software components in the information flow is crucial to identify the mechanisms generating specific genomic signatures. In this work, we perform a bibliometric analysis to identify the different purposes of looking for particular patterns in DNA sequences associated with a given phenotype. This study has enabled us to make a conceptual breakdown of the genomic signature and differentiate the leading applications. On the one hand, it refers to gene expression profiling associated with a biological function, which may be shared across taxa. This signature is the focus of study in precision medicine. On the other hand, it also refers to characteristic patterns in species-specific DNA sequences. This interpretation plays a key role in comparative genomics, identifying evolutionary relationships. Looking at the relevant studies in our bibliographic database, we highlight the main factors causing heterogeneities in genome composition and how they can be quantified. All these findings lead us to reformulate some questions relevant to evolutionary biology.

## 1. Introduction

Genomes are the physical entities that best record the history of life. Increasing evidence for the molecular mechanisms by which organisms evolve suggests that information plays a crucial role in life sciences. Novel mechanisms in data processing involve state transitions in biological systems and may be behind the origin of life and the major evolutionary transitions. In most prokaryotes and viruses, genomes are mainly composed of coding regions, and the genotype–phenotype mapping is one-to-one. However, most DNA mass is composed of non-coding regions in multicellular and complex organisms. These regions are characterized by repetitive sequences that provide structural and regulatory functions. Specific patterns related to the information encoded in DNA molecules are called Genomic Signature (GS). However, we must break this concept down depending on its categorical characterization. Here, we perform a conceptual review and differentiate genomic signatures at each level of the information flow. On the one hand, we identify a collection of signatures associated with a given phenotype, which we define as *gene signature*, *protein signature*, *mutational signature*, *immune signature,* and *molecular signature*. These signatures refer to expression profiles involved in a given biological function or metabolic pathway, such as antibiotic resistance or virulence. They focus on local properties in the genotype–phenotype mapping, crucial for genetic engineering and developing techniques in precision medicine. On the other hand, we use the term *selective signature* to denote the genotype-registering trait variation in populations that is subject to selective pressures. Finally, we use *organismal signature* to refer to the characterization of hidden patterns in DNA sequences, a global measure that identifies the organism involved. The organismal signature is at the core of alignment-free methods and is usually applied in comparative genomics and evolutionary studies.

In comparative genomics the organismal signature has established rigorous criteria to compare organisms based on molecular evidence. In traditional methods, inferring relationships is not as simple as looking at who resembles whom. Assuming that two similar sequences must have a close evolutionary origin can lead us to an incorrect reconstruction of the tree of life. The search for solutions to this problem leads us to the concept of homology, being the basis of the systematic sciences [1,2,3]. Homology refers to similar traits between biological entities due to their evolutionary ancestry [4]. On the other hand, homoplasy refers to similarities between phylogenetically unrelated species. Multiple alignment-based methods aim to identify the evolutionary relatedness among sequences according to their homology while discriminating homoplasy events. [5,6,7,8,9,10,11]. By 1990, these methods revolutionized the biology data-processing field [12]. However, most of these models have hidden assumptions that should be not overlooked. Among the most critical assumptions, we find the collinearity between sequences, i.e., that homologous sequences conserve a sequential order of the bases. Moreover, it is commonly assumed that different sequences evolve at the same rate, or that different regions in a sequence evolve independently from each other. Furthermore, most models are stationary, which implies that sequences reach a state of equilibrium with evolutionary time. It is even assumed that all sequences evolve under the same model. Some examples are the Jukes–Cantor model, where all nucleotide substitutions occur with the same probability [13], or the more realistic model of Kimura, referred to as Kimura-2P, in which transitions and transversions occur with a different probability [14]. With time, increasingly complex methods started to overcome some of these conditions [15,16,17,18,19,20]—for example, by including gap penalties [21], considering a heterogeneous distribution of mutation rates across point locations [22,23,24], assuming the non-stationarity [25], or accounting for heterotachy [26,27,28]. However, these complex methods started to approach a NP-hard problem, and new efforts were required to find an equilibrium between model complexity and its explanatory power. Phylogenetic reconstructions based on retroposons insertions illustrate this situation, where other complementary methods may be required [29,30]. For example, it may occur that not all inserted retroposons are fixed in a population before a speciation event, which could result in inaccurate ancestral reconstruction [29]. High-throughput sequencing and the development of new bioinformatic tools have facilitated the study of these repetitive elements. In particular, new phylogenetic methods based on abundances of repetitive DNAs have been developed to infer phylogenetic relationships of several plants and animals and to construct retroposon-based phylogenies [31,32,33,34].

Most challenges in comparative genomics were overcome by realizing that closely related organisms share similar abundances of word sequences, which motivated Karlin and his colleagues in 1995 to coin the term genomic signature as a measure of word frequencies able to differentiate species and identify evolutionary relationships [35,36,37]. Specifically, they found evidence that dinucleotide and tetranucleotide frequencies differentiate well between species. The discovery of characteristic patterns in DNA sequences gave rise to the so-called alignment-free methods, which find similarities at the genome level without the need for linear alignments or the presence of homologous sequences [12]. Here, these DNA patterns are what we call the organismal signature, which is on the basis of word frequency-free methods. The potential of the pairwise distance between organismal signatures was rapidly recognized and started to be largely applied in the literature [37,38,39]. Furthermore, in a recent publication the mapping of k-word distribution into a single value has been explored as a measure of organismal complexity [40]. Computing distance similarity among two given sequences consists of three basic steps. The first step consists of creating a library of k-words (i.e., oligomer sequences of length k) occurring along the DNA sequence. For example, the sequence ATTGCAT is composed of the following words of length k=2: {AT, TT, TG, GC, CA}, with *AT* occurring twice. The second step organizes k-word frequencies into an array, where each entry corresponds to the number of times each particular word of length *k* appears in the given sequence. Finally, the third step computes a metric to quantify the distance between two given word frequencies [39]. Thus, similarity is related to a distance metric, where two identical sequences would correspond to a distance length of zero.

This review is organized into three sections. In the first section, we perform a bibliometric analysis from all the literature where the concept of genomic signature acquires a specific meaning. We give an overview of the main fields of application and identify a proper definition for each case study. The second section reviews the so-called chaos game representation, a model for characterizing hidden patterns in genome sequences. We highlight the mathematical basis to define a measure of organismal signature. Finally, the third section reviews the most important findings in the literature when comparing organisms based on their organismal signature.

## 2. Bibliometric Analysis of the Genomic Signature

### 2.1. Methods

Bibliometric analysis is a method for analyzing the global structure of a research topic by looking at the relationships within bibliographic data [41]. In this review we have performed a bibliometric analysis of all articles where genomic signature appears as a focus of study, which has enabled us to differentiate its conceptual meaning depending on the research field of application. First of all, a bibliographic library was created from the Web of Science, one of the largest bibliographic databases. We have run a search for all articles where ‘genomic signature’ or ‘genome signature’ appears in the topic field, i.e., in the title, abstract, ID keywords, or author keywords, obtaining a total of 541 articles that span from 1994 to 2022 and were published in 280 different journals. Note that we have excluded review articles from the search. We have also generated a list with all keywords appearing in our bibliography database, corresponding to 2319 Keywords Plus (ID) and 1461 Author’s Keywords (DE).

Two different types of bibliometric analysis have been performed: co-word analysis [42] and bibliographic coupling [43].

In the first analysis, we have explored the structure of co-occurrence among keywords and identified the main fields of study linked to a genomic signature. We have considered the total of 3404 keywords, which decreases to 241 words by imposing a threshold of a minimum number of four occurrences. We have created a thesaurus file to clean the list of keywords manually. Specifically, we have merged all synonym terms and singular/plural relations. We have also clustered words referring to a specific type of cancer (i.e., ‘breast cancer ‘colorectal cancer ‘gastric cancer ‘ovarian cancer and ‘prostate cancer’ are clustered together and replaced by ‘cancer’). We have also merged the words ‘genomic signature’, ‘genome signature ‘signatures and ‘signature Instead, we keep ‘gene signature’ as a single word because it acquires a specific meaning in the literature. We have also merged the terms ‘genomes ‘genome ‘genetics and ‘genomics Not all words are biologically meaningful. Those unrelated to a biological concept are not of interest to us. So, we have removed non-relevant words that may be a source of noise in the network analysis (e.g., ‘American society ‘reveals ‘discovery ‘insights ‘features ‘subtype and ‘subtypes’). However, we do not exclude some words such as ‘identification’ or ‘diversity’ because we consider that they may play a role as key connectors linking closely related words. We produced a final list of 170 words from which we have conducted the co-word analysis. Specifically, we have generated a network where keywords correspond to nodes and where connections between words are weighted by the number of times they appear together as keywords in the literature. We identify the main themes where genomic signature is applied by looking at the clusters appearing in the network. Visualization of the network and the community detection algorithm are provided by the VosViewer software [44]. Here, we have considered only words appearing at least four times in the literature, and links are weighted by full counting and normalized by association strength.

The second analysis carried out is bibliographic coupling, a method to identify the main research lines where the genomic signature is applied and its evolution. It consists of a network where nodes represent articles and where links between two articles are proportional to the number of shared references. Thus, coupling strength is high for articles sharing similar bibliography. One advantage of this method is that connections are not influenced by the year of publication. Instead, recent publications have the same weight as earlier ones, so the network’s topological properties are informative about the evolution of a research topic and highlight the different lines of study. We built the network and ran the clustering algorithm provided by VosViewer software. In this case we have normalized links according to their association strength with fractional counting.

### 2.2. Co-Word Bibliometric Analysis

We identify the main topics in which genomic signature is applied by looking at the words that cluster together in the network. First, a library of words is prepared by manually cleaning the collection of keywords given in the literature, as described in Section 2.1. From our selection criteria, we performed the study with the 170 keywords appearing throughout the 541 articles. Table 1 shows a ranking list with the 24 most frequent words, together with their frequency (i.e., the number of times each word appears as a Keyword in our library) and the total link strength, which corresponds to the number of keywords with which a given word of our list appears together.

We observe some interesting results from this search. First, gene expression appears as the most abundant keyword in the literature, suggesting that genomic signature is highly linked to gene expression patterns. Looking at the period of published articles, we already observe the appearance of studies looking for gene markers around 2011, with words related to the field of health appearing throughout the full period of time. Among the most abundant words we find ‘cancer’, ‘chemotherapy’, ‘prognosis’, ‘differentiation’, ‘microarray’, and ‘biomarker’. However, the most abundant keywords appearing in the recent years are mostly associated to precision medicine, such as in cancer studies. On the other hand, abundant words such as ‘diversity’, ‘evolution’, ‘adaptation’, and ‘selection’ correspond to the field of evolutionary biology. Finally, the word ‘chaos game representation’, which is a mathematical model to characterize the structure of DNA sequences, appears overrepresented in the literature, highlighting its impact in comparative genomics.

**Figure 1:**
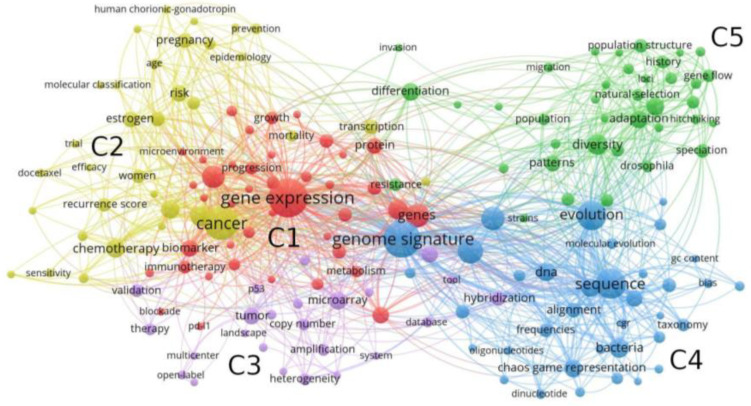
Co-word network characterizing the structure of keyword co-occurrences. The five clusters obtained from VosViewer are represented by colors and named as C1, C2, C3, C4, and C5.

We now identify the main word clusters according to their co-occurrence, as illustrated in Figure 1. We apply the community detection algorithm provided by VosViewer, finding a total of five clusters. Here, node sizes are represented according to their frequencies.

The two first clusters are associated with the health field, with ‘gene expression’ and ‘cancer’ appearing among the most abundant Keywords. This result is in agreement with the fact that genomic signature has a particular and important meaning in precision medicine. On the other hand, Cluster C3 is composed of words associated to the field of molecular biology. Genomic signature acquires a different interpretation in comparative genomics and evolutionary biology, with interconnected words composing clusters C4 and C5, respectively. ‘Frequency’, ‘alignment’, ‘sequence’, and ‘chaos game representation’ are some examples of words associated to genomic signature composing the field of comparative genomics, and ‘natural selection’, ‘adaptation’, and ‘diversity’ are related to evolutionary biology. Table 2 summarizes the identification of the four fields of study where genomic signature is applied.

### 2.3. Bibliographic Coupling

**Figure 2:**
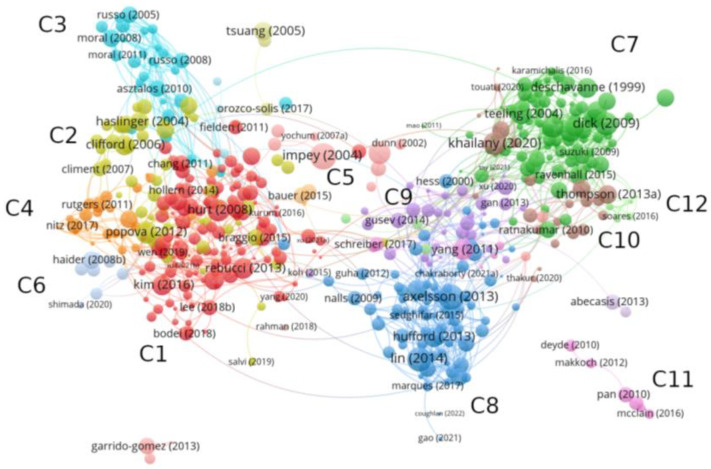
Bibliographic coupling of articles where ‘genomic signature’ appears as a Keyword. A total of 12 clusters is obtained from VosViewer, which are represented with colors and named as C1, …, C12.

A bibliographic coupling is carried out from all articles where genomic signature appears as a Keyword. As explained in Section 2.1, the clustering algorithm and visualization of the network has been provided by VosViewer, whose results are illustrated in Figure 2. We find a total of 12 clusters, which represent the topic fields where the genomic signature is applied.

A global view of the network shows a clear partition into two well-differentiated parts. The left-hand side of the network corresponds to applications in the health field, whose nodes are overrepresented by cancer studies. In general, genomic signatures in this part of the network are not associated to individual traits, but rather it informs about a given physical state that occurs under certain conditions (e.g., expression profile, presence of specific molecules). Nodes on the right-hand side of the network correspond to studies where genomic signature acquires a different meaning. On one hand, it is interpreted as a species-specific measure at the level of DNA sequences able to differentiate individuals according to their evolutionary history. This concept is more closely linked to a fingerprint of individual organisms. On the other hand, it is related to genome markers modulating variability in a population. In this case, it refers to DNA patterns in a population.

#### 2.3.1. Applications in Medicine

Looking at the most frequently cited papers within each cluster, we identify the different themes in which the genomic signature is applied. Cluster C1 is composed of 131 articles focusing on cancer studies. In [45], the authors analyze the genomic signature of prostate cells potentially involved in tumor development by identifying the expression patterns in a specific type of stem cell compared with the differentiated cells to which they give rise. This study has provided a better understanding of the behavior of cancer stem cells such as prostate-cell gene expression patterns, which are associated with a poor prognosis for cancer. These findings enable us to assess a patient’s prognosis and apply effective therapies. In [46], genomic signature refers to the biomarker that characterizes the resistance mechanisms of cancer cells to chemotherapy. Key mutations and gene expression profiles specific to each patient are then sought to establish action criteria adjusted to the resistance profile shown by the patient. Furthermore, the expression profiles in mutated genes that frequently appear in cancer are identified as genomic signatures of a potential factor of cancer [47]. Characteristic mutational signatures involved in cancer development are extensively analyzed in the literature associated with this cluster. These genomic signatures refer to expression profiles of a specific collection of genes with related activity or are associated to the common mutational pathways in tumors [48,49]. Cluster C2, which has 50 articles, combines studies that identify signatures associated with cancer origin and development, such as polymorphisms or key mutations. The study of genomic instabilities that play a crucial role in the development of human cancer is extensively covered in this cluster. Specifically, in [50], a genomic signature is defined to predict the instabilities of tumor suppressor genes, whose inactivation is commonly present in carcinomas. Meanwhile, [51] focuses on the molecular pathogenesis of active medulloblastomas. In [52], the authors analyze regions where differential gene expression occurs in chronic lymphocytic leukemia, and in [53], they focus on identifying a genomic signature in patients with colon cancer in stage II based on gene microarrays, which provides a good assessment of the patient’s prognosis.

Pregnancy produces a cascade of hormonal activity in the body and infers important changes in the breast. Most of the 30 articles composing cluster C3 focus on developmental disturbances in the breast during pregnancy, such as the effect of gene expression alterations [54] or prenatal exposure to certain organic compounds, such as the case of bisphenols [55]. A total of 27 articles about breast cancer gene signatures are collected in cluster C4. Most of these studies help to predict whether breast cancer will spread to other parts of the body by looking at the activity of a group of genes. Among the most cited articles within this group, we find a study identifying a 70-gene profile to establish clinical criteria that select patients for adjuvant chemotherapy [56]. Other studies develop a signature that predicts the response to trastuzumab, a drug widely used in treating breast cancer [57], or analyze the benefit of chemotherapy in breast cancer patients [58]. Transcript quantification, which identifies gene expression levels, is the theme grouping the 13 articles in cluster C5 [59,60]. Finally, cluster C6 is composed of eight papers. Although it presents a variable theme composition, some articles deal with skin pathologies [61,62].

From a global view of the content composing each cluster, we classify the concept of genomic signature in terms of their conceptual meaning. We refer to *gene signature* as the collection of genes involved in a specific function. It provides information about the activity of a specific gene group, which allows us to identify the origins and evolution of virulent strains, detect transmission flows in host–parasite relationships, or search for antibiotic resistance genes. Generally, gene signature is linked to a biological function and relies on the mechanisms by which genes activate or share properties among individuals. Notably, it also provides important information about cancer development. A related signature is the *protein signature*, which refers to gene expression profiling. It informs about the presence of expressed proteins in a specific location under specific conditions. It primarily identifies the treatment response and a patient’s prognosis. Another signature is the *mutational signature*, which corresponds to key mutations in the DNA that underlie the origin of cancer and share similar patterns across individuals. About 20 patterns have been discovered to yield most of the mutations present in common cancers. A fourth signature, which we call the *immune signature*, identifies the immunity response in a given host organism—studies referring to such a signature focus on identifying bacterial vectors for genetic engineering purposes. The immune signature provides information about the antibodies present in a given organism, as those in the human blood. In this case, signatures may change over time, which helps track a patient’s current state and make diagnoses. Finally, the *molecular signature* is an alternative term to the so-called biomarker. It tracks the presence of a particular molecule in the body and searches for its relatedness to a given disease or clinical condition. Analysis of treatment response is one of the main applications of molecular signatures.

#### 2.3.2. Applications in Comparative Genomics and Evolutionary Biology

Articles located on the right side of the network collect studies in the field of comparative genomics and evolutionary biology. Cluster C7 is the largest cluster, composed of 91 papers. Taxonomical classification [63] and phylogenetic analyses [64,65,66,67] are some problems addressed in this cluster. Other studies include the identification of intra-genomic and inter-genomic variations [37,38,39,68,69,70,71,72,73,74], codon usage biases in bacteria [75], and the classification of novel sequences obtained from metagenomic data [76,77,78,79]. It also collects studies analyzing host–parasite relationships [80,81,82,83,84,85,86,87] and evolutionary origins, such as in the case of SARS-CoV-2 and HIV. Finally, some studies are more related to the methodology used in comparative genomics, such as the search for species-specific genome patterns [88,89,90,91] and the development of theoretical measures able to highlight the hidden structure of genome sequences based on information theory [92,93] and higher-order Markov models [94].

The increasing interest in the molecular mechanisms driving the evolutionary history of species and the effect that adaptive selection has on genotype–phenotype mapping is reflected in Cluster C8. Here, genomic signature is strongly linked to the concept of hitchhiking, which assumes that selective pressures induce modifications in specific regions of the genome. The 82 articles composing this cluster are characterized by relevant studies in evolutionary biology, where the signature plays a key role in modulating phenotype characteristics in wild and domestic populations [95,96,97]. This perspective has motivated the search for signatures in populations of plants, animals, and humans. Cluster C9 is composed of 35 articles with a divergent focus of study. Among the most relevant studies we find the search for a genomic signature characterizing microbial communities [98,99], gene families associated with a biological function [99,100,101], horizontal gene transfer events contributing to the appearance of virulent strains [102,103], or the search for antibiotic resistance genes [103,104]. Cluster C10 comprises 24 articles focusing on the taxonomical classification of microbial communities [105,106], vibrio species [107], and pathogens of interest. Furthermore, this cluster captures several studies analyzing the origins and evolutionary history of SARS-CoV-2 [108,109]. Cluster C11 is composed of 13 articles, including studies about the evolution of diverse bacterial pathogens, host susceptibility [110,111,112,113], and signatures of influenza infections by pandemic viruses [114,115,116,117]. Finally, Cluster C12 is composed of eight articles, most related to the search for genomic islands, i.e., DNA fragments inserted into a genome through horizontal gene transfer [118,119,120,121,122].

Taking into account the primary goal of studies located in this network region, we can differentiate two types of genomic signatures relevant to evolutionary biology. We will use *selection signature* to denote such genomic regions capturing trait variability within a population. Finally, the *organismal signature* is proposed as any theoretical measure applied over DNA sequences to identify the phylogenetic relationship among biological entities. The frequency of oligonucleotides was initially proposed as an organismal signature [123], which has motivated the development of the so-called alignment-free methods in evolutionary analyses. In the following sections we focus on the organismal signature and explore its capacities and limitations.

## 3. Revealing Patterns in Genome Sequences

The discovery of specific patterns along DNA sequences was a starting point to quantify the organismal signature. It was in 1990 when Jeffrey applied the Chaos Game Representation (CGR) to DNA sequences and found evidence of hidden species-specific structures [123]. It was the first time in history that genome sequences were mapped into a visual representation, highlighting their local and global properties. In this section, we introduce the mathematical foundations of the method, which are adapted from [124].

### 3.1. Iterated Function Systems

An affine transformation in the two-dimensional space consists of a transformation $w\;:\;{\mathbb{R}}^{2}\to {\mathbb{R}}^{2}$ of the form:

(1) $$w\left(x_{1},x_{2}\right)\to \left(ax_{1}+bx_{2}+c,dx_{1}+ex_{2}+f\right),$$

where a, b, c, d, e, f∈ℝ are constant parameters. A more compact notation can be written with matrices:

(2) $$w\left(x_{1},x_{2}\right)=\left(\begin{array}{cc} a & b\\ c & d \end{array}\right)\left(\begin{array}{c} x_{1}\\ x_{2} \end{array}\right)+\left(\begin{array}{c} e\\ f \end{array}\right)=Ax+t,$$

where $A\in {\mathbb{R}}^{2\times 2}$ specifies the linear transformation and $t\in {\mathbb{R}}^{2\times 1}$ the translation. We are interested in one type of affine transformation, called contractive. In particular, the transformation w on the metric space $\left({\mathbb{R}}^{2},\;d\right)$, where d denotes de Euclidean distance, is a contraction mapping if

(3) $$d\left(w\left(x\right),w\left(y\right)\right)\leq s\cdot d\left(x,y\right)\;\;\;\forall x\in {\mathbb{R}}^{2},$$

for some constant 0≤s≤1. In the following, we will consider the particular case of s=1/2.

A (hyperbolic) iterated function system (IFS) is a finite set of contraction mappings ${\left\{w_{i}\right\}}_{1\leq i\leq m}$ defined on a complete metric space. We will focus on IFSs defined on the Euclidean plane with s=0.5, as in the example shown in Table 3. In this case, all contraction mappings reduce at half the initial compact set and displace it according to their respective translations.

Let $\left(ℍ\left({\mathbb{R}}^{2}\right),\;h\left(d\right)\right)$ denote the space of nonempty compact subsets of ${\mathbb{R}}^{2}$, with the Hausdorff metric. Then, by Theorem 7.1 of [124], the transformation $W:ℍ\left({\mathbb{R}}^{2}\right)\to ℍ\left({\mathbb{R}}^{2}\right)$ defined by

(4) $$W\left(X\right)=\overset{m}{\underset{i=1}{\cup }}w_{i}\left(X\right)\;\;\;\forall X\in ℍ\left({\mathbb{R}}^{2}\right)$$

is a contraction mapping on the complete metric space $\left(ℍ\left({\mathbb{R}}^{2}\right),\;h\left(d\right)\right)$. Starting from an initial compact set $X_{0}$, we can iteratively apply the transformation W as follows:

(5) $$\begin{array}{c} X_{1}=W\left(X_{0}\right)\\ X_{2}=W^{2}\left(X_{0}\right)=W\left(X_{1}\right)\\ \ldots \\ X_{n+1}=W^{n+1}\left(X_{0}\right)=W\left(X_{n}\right) \end{array}$$

**Figure 3:**
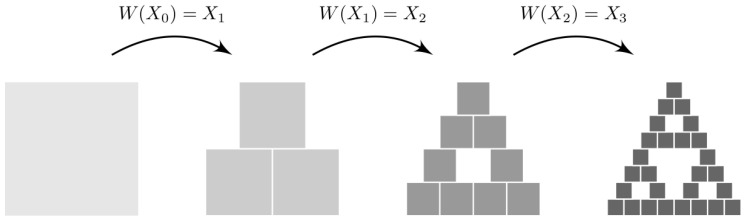
Iterative application of the contraction mappings of the system represented in Table 3 starting from an initial square box.

The transformation W has a unique fixed point, called the attractor of the IFS, and is given by $X^{*}=\underset{n\to \infty }{\lim}W^{n}\left(X\right)$. The fixed point fulfills $X^{*}=W\left(X^{*}\right)$. So, if we iterate the system from a random initial point, it will approach the attractor in a finite number of time steps from which it will never escape. As an example, Figure 3 shows the iterative application of the contraction mappings of the IFS described in Table 3 starting from an initial square box. If we continue applying W for a sufficiently large number of iterations, the Sierpinski triangle appears progressively.

The so-called chaos game refers to a method of creating fractals. We are interested in the random iteration algorithm [124], which assigns a probability distribution to the contraction mappings of the IFS. Thus, we create a sequence of points $\left\{X_{0},X_{1},\ldots ,X_{n}\right\}$ by iterating the map $X_{n+1}=w_{i}\left(X_{n}\right)$ from an initial point $X_{0}$, where $w_{i}$ is a member of the IFS randomly selected according to the probability $p_{i}$. The algorithm is a method of generating the attractor of any IFS, which has attracted many researchers due to its capacity to generate complex structures such as fractals. However, not all IFSs generate a fractal-like structure, such as the Sierpinski triangle. The system of Table 4 is composed of four contraction mappings, which map the initial box square $X_{0}$ into each of the four sub-quadrants, i.e., $W\left(X_{0}\right)=X_{0}$. Furthermore, the system has a uniform distribution assigned, so all contraction mappings $w_{i}$ have the same probability of application. As a consequence, the random iteration algorithm generates a sequence of points that are homogeneously distributed and no pattern appears.

The random iteration algorithm of the system of Table 4 can also be computed following this simple algorithm:

- 1. Take the four vertices (0, 0), (0, 1), (1, 0), and (1, 1) defining the unit square box.
- 2. Start from an initial random point in the unit square.
- 3. Select one vertex randomly, and compute the halfway point between the previous point and the vertex.
- 4. Repeat step 3 as many times as you want.

What happens if we now unbalance the assigned probabilities? In such a case, we would force the mapping machine to deviate from a pure, uniform random process. Even if we use a poor pseudorandom number generator in the mapping run, some heterogeneities will start to appear. Heterogeneities in point distribution arise from regularities in the iteration algorithm, which has led to the application of iterated function systems over sequences. Let us call the mapping sequence the consecutive sequence of contraction mappings in its order of application. Then, any regularity present in the sequence will be reflected in a visual pattern of the point trajectory. Now we have an algorithm able to capture the underlying patterns in a sequence. In particular, the graphical representation of sequences using iterated function systems was termed by Jeffrey, who applied it over DNA sequences, as the Chaos Game Representation (CGR) [123].

### 3.2. Underlying Patterns in DNA Sequences

**Figure 4:**
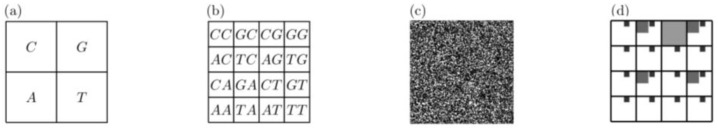
Visual representation of the Chaos Game Representation. (**a**) Sub-quadrants associated to the sequences of size k=1 and (**b**) k=2. (**c**) CGR of a random sequence. (**d**) Fractal-like structure of empty regions corresponding to the absence of the word “CG”.

The CGR assigns each nucleotide base to a contraction mapping $w_{i}$ of the IFS given in Table 4. However, now, instead of having probabilities associated to the mapping run, the rules are determined by the genome sequence. Thus, starting from a random initial point $X_{0}$ in the unit square, the CGR iterates the map $X_{n+1}=w_{i}\left(X_{n}\right)$ following the sequential order of the bases as they appear along a DNA sequence. We can also assign the four nucleotides to the corners of the unit square, A=(0,0), C=(0,1), G=(1,1), and T=(1,0) (Figure 4a). For RNA sequences, base T is replaced by U. The algorithm generates a new point halfway between the previous point and the corner associated with the next DNA base appearing in the sequence. There is a one-to-one relationship between sequences and point trajectories [125]. More specifically, if we divide the unit square into non-overlapping sub-quadrants of size 1⁄k×1⁄k, then each subsequence of length k corresponds to a unique sub-quadrant. For example, Figure 4b shows the corresponding sub-quadrants associated to the sequences of size k=2.

From this mathematical characterization, the CGR of a pure random sequence generates a uniform picture of dots, as illustrated in Figure 4c. On the other hand, regularities in the sequence generate a heterogeneous distribution of points with self-similarity properties. For example, Figure 4d shows the empty regions that replicate in smaller copies in the absence of the dinucleotide “CG” in a sequence (up to resolution k=4). So, the distribution of points highlights *k*-word abundancies and provides helpful information about the underlying structure of a sequence.

**Figure 5:**
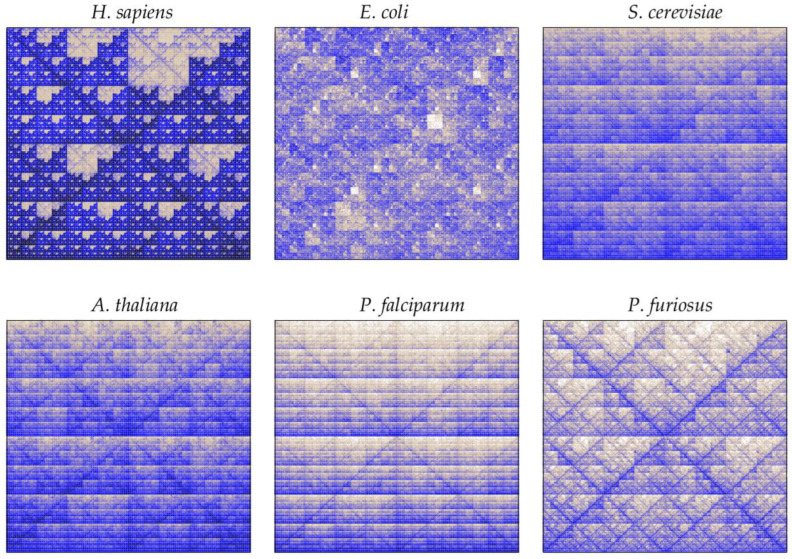
The $2^{8}\times 2^{8}$ FCGR images of genomic DNA sequences from *H. sapiens*, *E. coli, S. cerevisiae, A. thailana, P. falciparum, and P. furiosus.* The color map corresponds to word abundancies, i.e., the number of times each word of size k=8 appears in DNA sequences.

A quantized version of CGR is given by the frequency chaos game representation (FCGR_k_), which provides a $2^{k}\times 2^{k}$ matrix containing the frequency of all *k*-words in a DNA sequence [125,126,127]. Thus, heterogeneities in point distributions can be quantified by coarse-graining the unit square into $2^{k}\times 2^{k}$ regular boxes and computing the points’ density in each box. The FCGR_k_ displays an image in which each pixel is associated with a specific word of size *k*, and the intensity of the color map corresponds to word frequencies. Thus, the darker the pixel, the higher the word frequency. As an illustration, Figure 5 shows the FCGR of genomic DNA sequences from *H. sapiens*, *E. coli*, *S. cerevisiae*, *A. thailana*, *P. falciparum,* and *P. furiosus* for k=8.

The repetitive occurrence of a given word gives rise to a high density of points in the corresponding sub-quadrant. Dinucleotide abundancies of AT and GC are displayed as horizontal lines, as in the case of *S. cerevisae*, *A. thaliana,* and *P. falciparum*. On the other hand, translations and transversions place points along the diagonals, as we can observe in *H. sapiens*, *A. thailana*, *P. falciparum*, and *P. furiosus.* Self-similar patterns also occur, as in the case of the double-scoop in *H. sapiens*, illustrated as a fractal pattern of empty regions corresponding to an absence of the CG dinucleotides. Fractality, instead of appearing due to the non-randomness of sequences, is a direct consequence of the conserved statistical properties of *k*-words when increasing its size *k*. The presence of a given word implies that at least one word of larger size contains it. Thus, word frequencies constrain the distribution at larger word sizes. It also implies that the presence of regular patterns in DNA sequences appears as a fractal-like structure. In this case the fractal structure is associated to the absence of “CG”, such that no point can fall inside the regions associated to mapping sequences that contain the “CG” word.

Although the CGR is an important starting point, it only provides a qualitative picture of the underlying patterns. We need additional tools to find a measure able to quantify the observed patterns. For example, one could be interested in looking at the resolution that maximizes the variability of word frequencies, which intuitively corresponds to the length scale of words that would optimize the information encoded, but other quantities may be of general interest, such as the presence or absence of specific words.

## 4. Genomic Signature in Evolutionary Biology

Evidence of species-specific patterns in DNA sequences started in the early 1960s, when a biochemical experiment showed that relative dinucleotide abundance is a stable property of DNA sequences [128]. However, owing to the scarce data available in the following 30 years, it was not until 1990 that significant conclusions started to be drawn. The comparison of DNA sequences supported the hypothesis that word frequencies follow an evolutionary history, which led Karlin et al. to conclude the existence of an organismal signature [35].

Based on the increasing empirical evidence, dinucleotide relative frequencies were initially proposed as a proper signature describing the inter- and intra-genomic variations, which is mathematically defined as follows:

(6) $$\frac{f\left(XY\right)}{f\left(X\right)f\left(Y\right)}$$

where X and Y denote nucleotide bases and f the frequency. Similarities between DNA sequences are commonly quantified by computing a distance metric over word frequencies. The initially proposed Euclidean metric is defined as follows:

(7) $$\delta \left(A,B\right)=\frac{1}{n}\sum^{\;}\left|f_{A}\left(XY\right)-f_{B}\left(XY\right)\right|\;$$

where A and B denote the sequences under comparison and n=16 corresponds to the total number of words of size k=2. Other metrics have also been used for this purpose, such as the Pearson correlation distance [129], the DSSIM [130], the Manhattan distance [131], or the approximated information distance [39]. From this characterization, the succession of nucleotides along a sequence follows a zero-order Markov chain, i.e., the probability of finding a given nucleotide does not depend on its neighbor composition. Thus, the probability of finding a word is the product of the probabilities of its constituent letters.

A generalization of *k*-word frequency distribution to any length enables us to address the problem in a more realistic framework. As we noticed before, each DNA sequence may be characterized by a length scale given by the word size at which the variability of word frequencies is maximized. However, there are some unsolved questions. Can we classify biological entities depending on such a characteristic length? Does it depend on the genome size? Regarding this last question, there is some evidence that this is not the case. For example, genome duplication mechanisms increase genome size while maintaining relative word frequencies as invariable. In order to find an optimal word size characterizing word frequency, we can establish some criteria based on statistical laws. In random sequences, the entropy of word frequencies is maximized for word sizes $k=\log_{4}\left(\left|s\right|\right)$, with |s| denoting the whole sequence size [132]. It means that we expect to find each word of size *k* only once along the sequence. For example, if we have a genome composed of 1 million bases, from a uniform distribution, we expect to find a frequency of one for each word of size k=10. As a consequence, this word size is an upper bound from which deviations from a uniform distribution would be observed with some significance. Furthermore, as more statistical significance is desired, lower word sizes would be required. A common approach when comparing different sequences is to fix a word size according to some prior. Because empirical evidence suggests that closely related sequences will share similar word frequencies, the deeper the taxonomic relationship is, the larger the word sizes will be required to be in order to differentiate their DNA sequences. So, the word size is usually fixed at values smaller than *k* but kept sufficiently large depending on the taxonomic level under study. It also may happen that a study searches for unique sequences. For example, if we search for a specific sequence in a genome of 1 Mb, one may argue that a word size of k=13 would be very convenient in order to keep an error percentage less than 2%. However, in most cases, the word size is usually fixed arbitrarily, highlighting the lack of a formal theory to compare sequences.

### 4.1. What Is Causing the Organismal Signature?

When looking at the distribution of word frequencies, the usual situation is that most of the words never appear along the DNA sequence, some appearing only once (mainly corresponding to genes) and a few being overrepresented (primarily associated with structural functions). For example, a case study in *A. fulgidus* using a word size of k=8 shows that a few words are very abundant, while about 300 words appear once at the most [37]. Which factors are modulating the non-randomness in genome sequence?

Different studies provide evidence of robust intra-genomic variability. They suggest that these patterns are driven by two main mechanisms: selective pressures subject to environmental conditions and specific processes associated with the genetic machinery, such as DNA replication and repair-based mechanisms. A case study in prokaryotes shows that oligonucleotide usage variability, AT content, phylum, and oxygen requirement are the main factors contributing to long-term intra-genomic patterns [70]. A direct consequence is that higher biases in nucleotide usage generate a more robust signature. GC content’s variability in microbes has also been associated with replication activity [133,134]. A study shows that genomes rich in GC content are more homogeneous than AT-rich genomes [76]. However, genomic signatures in prokaryotes based on dinucleotide abundances do not correlate to environmental conditions, such as habitat resources, osmolarity, and chemical conditions [72]. On the other hand, codon signatures show that codon usage is independent of GC content, gene size, and transcriptional and translational constraints but, rather, is related to the replication and repair process [135]. Thus, similarities in genome composition are partly explained because closely related organisms share similar proofreading mechanisms. They can modulate the variability of dinucleotide abundances (i.e., GC content) or amelioration in bacteria.

### 4.2. Dinucleotide Biases

Single nucleotides are not equally distributed along the genome, i.e., we do not find 25% of each base in each genome. In turn, nucleotide usage has a bias, which modulates the organismal signature. Relevant findings from comparisons of base abundances are related to AT and GC contents, where the proportion of guanine and cytosine along DNA sequences is referred to as the GC content. Similarities in CG depletion are observed in some eubacteria, archaebacteria, and eukaryotes. Some bacteria and archaea share an underrepresentation of CTAG. However, the abundance of words varies from one species to another [136,137]. In humans, GC content is about 40%, whereas in *Plasmodium falciparum*, GC content is about 20% (it is an AT-rich genome). Specifically, it has been found that DNA sequences rich in GC content show a more homogeneous genomic signature if compared to AT-rich genomes, in part due to a mutational bias in AT-rich genomes [137]. The energetic cost of having a GC-rich composition is higher than AT-rich dinucleotides, but it provides more stability to genomes [138]. The stability provided by a high GC content is due to the molecular interactions throughout the base stacking of adjacent bases. However, it is not clear what the specific advantages of GC-rich genomes are, nor what interspecies differences exist. For example, although it confers high stability to DNA molecules, in some bacteria with high GC content, autolysis has been found to occur easily. Furthermore, because sequences with abundant GC content confer higher thermostability, it was previously assumed that this bias is a consequence of an adaptation to thermal conditions. However, this hypothesis is no longer supported by empirical evidence. In turn, variations in GC content in more complex organisms show a mosaic pattern, shaping the so-called isochores. These regions are compositional domains of more than 300 kb with a homogeneous presence in GC content and are the main factor causing intra-genome variability [133,139,140]. The formation of these compositional domains is linked to multiple biological variables, such as gene density, replication rates, timing, and recombination [134]. However, their presence can vary in organisms of the same species. Despite the isochores, the overall distribution of dinucleotides is homogeneous throughout the genome when comparing pieces of 50 kbp. However, while dinucleotide abundancies among coding and noncoding regions do not show significant variation, it has been found that tetranucleotides differentiate these two regions. Dinucleotide and tetranucleotide biases in prokaryotes are analyzed in [74]. Moreover, stop codons are biased towards AT content, so the presence of genes may influence these biases.

Studies analyzing dinucleotide biases in the different kingdoms of life show that prokaryotes have an underrepresentation of dinucleotides. Most eukaryotes have an underrepresentation of AT-content, while some organisms such as insects, worms, and most fungi have typical CG values. On the other hand, GC content is overrepresented in many bacterial genomes [72]. Di- and tetranucleotide abundances effectively discriminate DNA sequences from different phyla [35]. A study comparing species from different domains of life reveals that the highest variability of dinucleotides among eukaryotes, bacteria, and archaea correspond to the AT-rich content, i.e., A + T is the main factor describing the variations among genome sequences [37]. However, while nucleotide concentration characterizes species, it does not differentiate organisms at high taxonomic levels. For example, mammalian species have independently undergone an increase in GC content, mainly due to the structure of genes and GC-biased gene conversion.

### 4.3. Taxonomic Inference from Word-Based Metrics

Genome composition remains robust throughout the whole genome, suggesting that genome-wide comparisons do not provide more information than using only small pieces of the DNA chain. Many studies have found evidence that intragenomic distances are smaller if compared to genomes from different species [37,39]. In microbial genomes, word frequencies have been shown to be similar when considering smaller fragments thereof, measuring about 10–50 kbp [74]. In bacterial genomes, intragenomic patterns are also found to vary less than intergenomic comparisons.

Word-based methods are at the core of the alignment-free methods and are receiving increasing attention in the scientific community [141]. A variety of case studies perform comparisons of word frequencies between organisms [64,69,142]. Comparisons of sequences representing all kingdoms of life are given in [39]. In this study, authors select sequences within a given chromosome of *H. sapiens* (animalia), *S. cerevisiae* (fungi), *A. thailana* (plantae), *P. falciparum* (protista), *E. coli* (bacteria), and *P. furiosus* (archaea), and they compute pairwise distances between genomic sequences using words of size *k* = 9. The method can classify all genomic sequences correctly, even at lower taxonomic levels. In this last case, comparisons are performed among *H. sapiens* (class Mammalia, order Primates) and *Mus musculus* (class Mammalia, order Rodentia). However, the authors highlight that the application of a metric should depend on the type of study and the taxonomic level of interest. Intragenomic patterns also display higher similarities than genomes from different species, supporting the existence of a species-specific organismal signature. Furthermore, the interrelationship among a large dataset of 3.176 mitochondrial genomes is analyzed in [38]. A Molecular Distance Map using DSSIM distance of words of length k=9 organizes the different taxonomic categories into non-overlapping clusters, with few exceptions. The study is applied to mtDNA sequences within Vertebrata, the superkingdom Protista, and the classes Amphibia, Insecta, and Mammalia. All genomic distances successfully classify the different sequences into their taxonomic categories. It is interesting to recall that a few sequences overlap within two different clusters but generally correspond to sequences whose classifications are still ambiguous. For example, within the subphyla of jawed vertebrates, they observe two fish species with primitive pairs of lungs, *Polypterus ornatipinnis* and *Polypterus senegalus*, converging in the cluster of amphibians. Finally, the compositional characteristics of metagenomic data also allow taxonomic labels to be assigned to individual genome sequences, classify unknown organisms, and assess microbial community profiling [65,77]. Multiple unsupervised clustering and metagenomic binning methods are also developed to find meaningful semantic clusters [143,144,145,146].

One important aspect in comparative analyses based on *k*-word frequencies is the presence of repetitive sequences, which has recently been discussed in the literature. These elements occur in multiple copies throughout the genome in higher plants and vertebrates and cover up to 65% of human genome. As a consequence, they contribute largely to the organismal signature. Different studies point out that the presence of repetitive elements contribute to the phyogenetic signal [32,147,148,149]. In [147], authors use an alignment-free method based on k-words of distinct genomic regions to infer the phylogenetic tree of Symbiodiniaceae. While different genomic regions, such as genic and non-genic regions, exhibit distinct phylogenetic signals, the results indicate that whole-genome data are the best choice for phylogenetic reconstructions. In concordance with this statement, reconstructions using specific regions may be useful to investigate different selective pressures during evolution [148].

### 4.4. Examples of Case Studies

#### 4.4.1. Horizontal Gene Transfer

Alignment-free methods based on word frequencies also provide important information about horizontal gene transfer events. In [71,84], horizontal gene transfer in bacteria is analyzed by looking at biases in dinucleotide composition. Remarkably, this study compares heterogeneities in genome sequences based on GC composition and finds anomalies in essential genes. On the other hand, plasmids are generally transferred laterally among bacterial cells and use the host machinery to replicate and obtain new copies. Dinucleotide abundances have resulted in minimal distances between plasmid sequences and their hosts in prokaryotes [72].

#### 4.4.2. Phage–Host Relations

The wide diversity of phages in nature is extraordinary, these being the most abundant organisms on Earth. The absence of homology in phages and increasing evidence about their mosaic structure have limited the characterization of phages for a long time. They have mainly been classified in terms of their nature and morphology (e.g., characteristics of their viral capsid). However, the assumption that related phages share common traits is no longer supported. Phenotype traits are not enough to explain the lifestyle of phages or to determine the phage cycle (i.e., if it is lytic or temperate). To solve this problem, studying phage–host relationships may open new insights. In [80], authors use the organismal signature to obtain phage–host relationships and determine if they are lytic or temperate. The comparison is performed by looking at the Euclidean distance of genomic signatures between each phage and the infected host. For the study, they use phages belonging to the Caudoviridae family and compare them to the four infected bacteria. The first result shows that phages and hosts have a broad spectrum in base composition. Specifically, they find that *E. coli* has a GC content of 50.8%, *P. aeruginosa* 66.6%, *S. aureus* 32.8%, and *M. smegmatis* 67.4%. Organismal signatures are computed using tetranucleotide frequencies. However, standardization is performed due to the large difference in nucleotide base composition. It is important to recall that distances here do not correspond to distances between phages, but their closeness is associated with a similar distance to their host. The Euclidean distance between bacteriophages and their hosts reveals an empirical threshold separating temperate vs. lytic phages. Temperate phages can integrate their genomes into the host, resulting in a shorter distance to the host if compared to lytic phages, which are located on the other side of the threshold. Summarizing, the representation of these distances in a one-dimensional space effectively separates phages with different lifestyles. However, families overlap within this distance-to-host dimension. The genome length, which has a non-homogeneous distribution among phages, is used as a second dimension, resulting in phage family clustering.

#### 4.4.3. Phylogenetics and SARS-CoV-2

More recent studies analyze the origin and possible recombination of SARS-CoV-2. In [150], a possible recombination of SARS-CoV-2 with Pangolin and Bat coronavirus is analyzed by looking at the Frequency Chaos Game Signal (FCGS), a method to detect hidden periodic signals in *k*-word frequencies. They find that SARS-CoV-2 is more closely related to Bat, with 96% of genome identity. However, intra-genomic variations show that Pangolin has the highest nucleotide identity in the S gene sequence, which suggests a possible evolutionary origin from Bat and Pangolin strains. A more sophisticated method is used in [109]. The focus of this study is to identify the origin of SARS-CoV-2 from a machine learning algorithm able to classify unknown sequences at each taxonomic level. The training dataset is based on the organismal signature of about 5000 unique viral sequences, including Bat Betacoronavirus. As they notice, moving down into the taxonomic levels implies that sequences are much more similar, so they justify that k=7-word sizes are large enough to compare closely related sequences. This approach has supported the hypothesis that the sub-genus Sarbecovirs and Betacoronavirus originated in Bat.

## 5. Discussion

First, we have performed a bibliometric analysis of the role of genomic signatures in biology. We have collected all articles where genomic signature appears as a Keyword and performed both a co-word analysis and a bibliographic coupling. By looking at the articles that cluster together in the bibliographic coupling, we identified the different fields of application and broke down the conceptual meaning of the genomic signature. We have used the term *gene signature* to denote the collection of genes involved in a specific biological function, *protein signature* for gene expression profiling, *mutational signature* for key mutations yielding a specific biological state, *immune signature* for the immune response within a host, and *molecular signature* for a biomarker. On the other hand, we have used *selection signature* to refer to the genomic regions registering the trait variability in a population and *organismal signature* for any measure able to identify phylogenetic relationships.

In the second part of the article, we have reviewed the formulation and applications of the organismal signature. We have looked at the mathematical foundations of the so-called Chaos Game Representation (CGR) and its applicability to genome sequences. It was in 1990 when Jeffrey showed a visual representation of the underlying patterns of DNA sequences, highlighting large biases in word frequencies. Furthermore, the comparison of CGR’s pictures among different species has provided new insights into the search for a species-specific measure. Specifically, distance metrics comparing word frequencies show low intra-genomic variability if compared to DNA sequences of distantly related species. Furthermore, increasing empirical evidence supports the hypothesis that word frequencies represent a conserved property in evolution, such distances among genome sequences are in concordance with evolutionary relationships. These results have placed word statistics at the core of the alignment-free methods in comparative analysis.

In recent decades the organismal signature has been applied to different problems of evolutionary biology. It has successfully classified species spanning all kingdoms of life, even at high taxonomic levels. For the first time, it has been possible to classify unknown sequences from metagenomic data and identify horizontal gene transfer events efficiently. Furthermore, it has contributed to a better understanding of phage–host relationships. For example, the distances of phages to their hosts have revealed phage lifestyle, i.e., whether they are lytic or temperate. Finally, the phylogenetic origins of certain sequences of interest have been determined, such as the case of SARS-CoV-2 and HIV.

Despite the advantage of the word-based alignment-free methods, the development of a rigorous formulation describing a quantitative measure of organismal signature is still lacking. We have formulated the hypothesis that the characteristic length scale of DNA sequences may be given by the value of *k* that maximizes the variability of *k*-word abundances. However, further studies should be conducted to find a mathematical solution to this problem and identify an optimal *k*-word length in alignment-free methods. Furthermore, there are still open questions. In case such a characteristic length exists, how does it relate to the genome size? Could it be informative about the genome complexity? How does it determine the optimal length for comparing different sequences? Moreover, it may be important to explore its limitations. For example, *k*-word frequencies do not take into account the relatedness among neighbor words, or the presence of long-range correlations throughout the genome sequences. It may also happen that sequences are characterized by different length scales according to their structural and functional fates. In such a case, a partition into compositional domains may better describe the informational properties encoded in genomes.

## Figures and Tables

**Table 1:** The first 24 most common Keywords, number of occurrences, and total link strength.

Keyword	Frequency	Total Link Strength
Gene expression	141	570
Genome signature	125	530
cancer	106	468
sequence	81	317
evolution	72	316
identification	52	219
genes	45	194
genome	45	170
cells	40	150
diversity	33	117
survival	31	135
mutation	29	106
bacteria	27	125
chaos game representation	26	117
chemotherapy	26	123
DNA	26	106
*Escherichia coli*	25	123
prognosis	24	122
differentiation	22	85
microarray	22	105
adaptation	21	90
selection	21	81
biomarker	20	90
phylogenetic analysis	20	94

**Table 2:** Classification of clusters given by VosViewer.

Scientific Field	Cluster	Num. Keywords
Health	C1	41
	C2	34
Molecular biology	C3	24
Comparative genomics	C4	36
Evolutionary biology	C5	38

**Table 3:** IFS composed by the contraction mappings $w_{1},\;w_{2}$ and $w_{3}$.

w	a	b	c	d	e	f
$w_{1}$	1/2	0	0	1/2	0	0
$w_{2}$	1/2	0	0	1/2	1/2	0
$w_{3}$	1/2	0	0	1/2	1/4	1/2

**Table 4:** IFS composed through four contraction mappings with associated probabilities *p*.

w	a	b	c	d	e	f	p
$w_{1}$	1/2	0	0	1/2	0	0	1/4
$w_{2}$	1/2	0	0	1/2	0	1/2	1/4
$w_{3}$	1/2	0	0	1/2	1/2	1/2	1/4
$w_{4}$	1/2	0	0	1/2	1/2	0	1/4

## Data Availability

Not applicable.
